# Impact of brain tumors and radiotherapy on the presence of gadolinium in the brain after repeated administration of gadolinium-based contrast agents: an experimental study in rats

**DOI:** 10.1007/s00234-019-02256-3

**Published:** 2019-07-11

**Authors:** Gregor Jost, Thomas Frenzel, Janina Boyken, Hubertus Pietsch

**Affiliations:** Bayer AG, MR & CT Contrast Media Research, Muellerstrasse 178, 13353 Berlin, Germany

**Keywords:** Gadolinium, Contrast agents, Magnetic resonance imaging, Brain, Blood-brain barrier

## Abstract

**Purpose:**

To investigate the impact of blood-brain barrier (BBB) alterations induced by an experimental tumor and radiotherapy on MRI signal intensity (SI) in deep cerebellar nuclei (DCN) and the presence of gadolinium after repeated administration of a linear gadolinium-based contrast agent in rats.

**Methods:**

Eighteen Fischer rats were divided into a tumor (gliosarcoma, GS9L model), a radiotherapy, and a control group. All animals received 5 daily injections (1.8 mmol/kg) of gadopentetate dimeglumine. For tumor-bearing animals, the BBB disruption was confirmed by contrast-enhanced MRI. Animals from the tumor and radiation group underwent radiotherapy in 6 fractions of 5 Gray. The SI ratio between DCN and brain stem was evaluated on T1-weigthed MRI at baseline and 1 week after the last administration. Subsequently, the brain was dissected for gadolinium quantification by inductively coupled plasma-mass spectrometry. Statistical analysis was done with the Kruskal-Wallis test.

**Results:**

An increased but similar DCN/brain stem SI ratio was found for all three groups (*p* = 0.14). The gadolinium tissue concentrations (median, nmol/g) were 6.7 (tumor), 6.3 (radiotherapy), and 6.8 (control) in the cerebellum (*p* = 0.64) and 17.8/14.6 (tumor), 20.0/18.9 (radiotherapy), and 17.8/15.9 (control) for the primary tumor (*p* = 0.98) and the contralateral hemisphere (*p* = 0.41) of the cerebrum, respectively.

**Conclusion:**

An experimental brain tumor treated by radiotherapy or radiotherapy alone did not alter DCN signal hyperintensity and gadolinium concentration in the rat brain 1 week after repeated administration of gadopentetate. This suggests that a local BBB disruption does not affect the amount of retained gadolinium in the brain.

## Introduction

Gadolinium-based contrast agents (GBCAs) are frequently used in clinical MRI examination in particular for CNS imaging. In the last years, an increasing number of studies reported increased signal intensities (SIs) on unenhanced T1-weighted images in the dentate nucleus (DN) and the globus pallidus (GP) of patients that received repeated contrast-enhanced MRI examinations [1, 2]. These signal hyperintensities depend on the ligand type of the agent [3, 4] and has been primarily observed after repeated administration of linear GBCAs [1, 2, 5] and not after macrocyclic GBCAs [6] which possess higher in vivo stability through their kinetic inertness [7].

Systematic animal studies confirmed and complimented the different findings for linear and macrocyclic GBCAs. In addition to the MRI SI changes [8, 9], these studies provided basic data about the brain gadolinium (Gd) concentrations [10, 11] and its distribution [12, 13], the long-term excretion from the brain [13, 14], and the chemical speciation of the Gd present in the brain [15, 16]. Animal studies also led to a better understanding of the pathway for traces of GBCAs from the blood into the brain tissue and identified the blood-cerebrospinal fluid (CSF) barrier as an initial point of entry [9]. It was demonstrated in healthy rats that small amounts of GBCASs can cross the blood-CSF barrier independent of their chemical structure or physicochemical properties [17]. Recently, this was confirmed in a clinical study where Gd was detected in the CSF even in patients with presumable intact blood-brain barrier (BBB) [18].

Following intravenous injection, GBCAs cannot pass the intact BBB [19], but enhance areas with a pathologically disrupted BBB, which is a major indication for contrast-enhanced MRI [20]. The influence of a pathologic or treatment-related interruption of the BBB integrity on the signal hyperintensity on unenhanced MRI and the Gd presence in the brain after repeated GBCA injection is widely unknown and controversial. In retrospective patient studies, the status of the BBB integrity and function during repeated contrast-enhanced MRIs is either not known or can be most likely characterized as a local BBB disruption. It may be assumed that different disease processes and stages lead to a large variability in the BBB status in these patient studies. Also, the treatment with radiotherapy can impact BBB permeability, particularly at an early phase after irradiation [21, 22]. The role of radiotherapy on the SI changes after repeated GBCA administration was addressed in several studies. Clinical observations in children found that T1 hypersignalintensities in DN and GP occurred more frequently in patients who underwent radiochemotherapy than in patients without this treatment [23]. In several adult patient studies, statistical analysis revealed no significant effect of radiotherapy on SI changes in DN and GP [4, 5, 24]. It was also reported that MRI SI alterations in the DN has been found after radiotherapy independent of GBCA administration [25, 26].

To date, the impact of BBB integrity on the Gd presence in the brain after repeated GBCA-enhanced MRI is not yet fully understood. The purpose of this study was to evaluate the impact of BBB disruption on the presence of Gd in the brain and on T1-weighted MRI SI changes after repeated administration of a linear GBCA in an experimental rat model of brain tumor and radiotherapy.

## Material and methods

### Animals

For this study, 18 Fischer rats (F344/DuCrl) were obtained from Charles River (Sulzfeld, Germany). The animals were kept under standard laboratory conditions at a temperature of 22 °C and a dark/light rhythm of 12 h. Standard rat chow and water were provided ad libitum. The animals were handled and treated according to German animal regulations. MRI and radiotherapy were performed under anesthesia with 1.5–2% isoflurane (Baxter GmbH, Unterschleißheim, Germany). The tumor cell inoculation was done under general anesthesia introduced by a mixture (1+2) of xylazine hydrochloride (20 mg/mL, Rompun, Bayer Vital GmbH, Leverkusen, Germany) and ketamine hydrochloride (100 mg/mL, Ketavet, Pfizer, Pharmacia GmbH, Berlin, Germany) of 1 mL/kg body weight intramuscular injected.

### Study setup

**Fig. 1:**
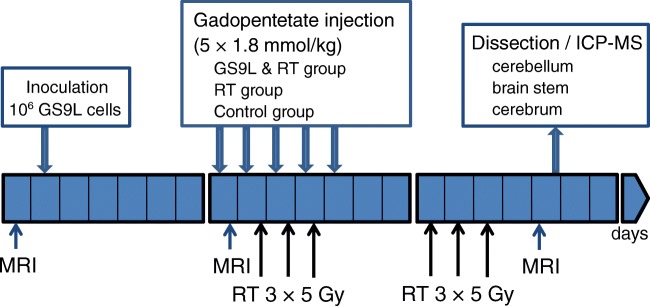
Study setup. GS9L cells were inoculated into the cerebrum of animals from the tumor group. At day 8, the presence of the tumor was confirmed by MRI. All animals received 5 intravenous injections (each 1.8 mmol/kg) of gadopentetate dimeglumine (day 8–12). T1-weighted brain MRI was performed for all animals at day 1 and 1 week after the last GBCA administration. Tumor-bearing animals (GS9L and RT group) and healthy animals from the radiotherapy (RT) group underwent radiation therapy (RT) in 6 fractions of 5 Gray on day 9–11 and 15–17. After the last MRI, the brain was dissected for Gd quantification with inductively coupled plasma-mass spectrometry (ICP-MS)

The animals were randomly divided into 3 groups (*n* = 6 each) and a baseline T1-weighted brain MRI was performed on all groups at day 1. In group one (GS9L and radiotherapy group), GS9L tumor cells were inoculated into the cerebrum of animals. The tumor presence was confirmed 1 week after inoculation by MRI and radiotherapy was started. The animals underwent radiation therapy with total dose of 30 Gray applied in 6 fractions of 5 Gray on day 9–11 and 15–17. The second group (radiotherapy group) consisted of healthy animals, which received an identical radiotherapy treatment. A third group (control group) of healthy untreated animals served as control. All animals received 5 intravenous injections (each 1.8 mmol/kg, cumulative dose = 9 mmol/kg) of linear gadopentetate dimeglumine (Magnevist, Bayer Vital GmbH) on day 8–12. Gadopentetate was administered by hand injection (~ 0.8 mL/min) in the morning without anesthesia. The time between injection and subsequent radiotherapy was at least 2 h. For the evaluation of signal enhancement of the DCN, a final brain MRI was performed at day 19, corresponding to 1 week after the last GBCA administration. Subsequently, the animals were euthanized and the brain was dissected for Gd quantification with inductively coupled plasma-mass spectrometry (ICP-MS). A schematic summary of the study setup is shown in Fig. 1.

### Tumor model

**Fig. 2:**
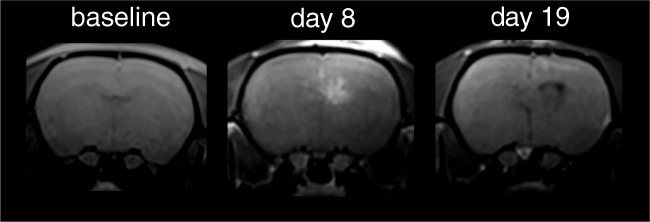
MR images before GS9L cell inoculation (baseline) 1 week after inoculation (day 8) and at the end of the study (day 19). On day 8, the tumor showed a strong signal enhancement 1 h after the administration of gadopentetate dimeglumine. An increased lesion size and a midline shift were found on the MRI acquired 1 week after the last administration (day 19)

The GS9L cell line was grown in Dulbecco’s Modified Eagle Medium (DMEM) supplement with 10% fetal bovine serum and 1% Penicillin-Streptomycin. For orthotopic intracerebral implantation, anesthetized animals were fixed in a stereotactic apparatus. A small hole was drilled trough the scull (Schick GmbH, Schemmerhofen, Germany) 2 mm anterior to the bregma and 2 mm dexter to the midline. Subsequently, 1.0E+06 GS9L cells suspended in a volume of 5 μL medium were injected slowly into the right hemisphere of the cerebrum using a Hamilton syringe. One week after cell inoculation, the animals underwent MRI to confirm the presence of the tumor. Therefore, a T1-weighted brain MRI was performed 1 h after the GBCA administration on day 8 (Fig. 2).

### Radiation therapy

Irradiation was performed at 140 kV by a computed tomography (CT) unit (Somatom Definition, Siemens Healthcare GmbH, Erlangen, Germany). For radiotherapy, the CT system was calibrated to the absorbed dose to water using an ionization chambers (PTW 31010, PTW Freiburg, Germany) placed at the isocenter of the gantry. A 3-mm PMMA cap was used to simulate the absorption of the rat skull. The CT was operated without table feed and a collimation 10 mm that results in a large in-plane field (50 × 50 cm) and a well-defined 1 cm radiation field in cranial-caudal direction. The CT tube voltage was 140 kV and tube current time product was adjusted to 265 mAs. This result in a dose of 5 Gy after 100 repetitive scans. The time delay between the scans was 5 s, and total time for irradiation was 500 s. For radiotherapy, the anesthetized rat was place in prone position and the head was positioned centrally at the isocenter using the laser positioning system and anatomical landmarks. The animal eye was positioned just below the level of the horizontal laser line. The transversal line of the laser representing the center of the radiation field in cranial-caudal direction was placed at ¼ of the distance between the base of the ear and eye.

### MRI protocol

For MRI, a clinical 1.5 T unit (Avanto fit, Siemens Healthcare GmbH) with a 2-channel rat head coil (Rapid Biomedical GmbH, Rimpar, Germany) was used. After a localizer scan, a 3D T1-weigted TSE sequence (TR = 500 ms, TE = 19 ms) was adjusted to cover the brain with a resolution of 0.3 × 0.3 × 0.8 mm (FOV = 80 × 32.5 mm; 30 transversal slices). A turbo factor of 3 was used; the number of signal excitations was 1 and the acquisition time was 9:55 min.

### Image evaluation

A quantitative image evaluation was performed blinded to the experimental groups on an external workstation (SyngoVia, Siemens Healthcare GmbH). Manually defined regions of interests were drawn around the deep cerebellar nuclei (DCN) and the brain stem guided by anatomical landmarks taken from a stereotactic rat brain atlas as described previously [9, 27]. The SIs for the left and right hemisphere were averaged and the DCN-to-brain stem SI ratio (DCN/brain stem) was calculated by dividing the mean SI of the DCN by that of the brain stem.

### Tissue processing and quantification of Gd

The brain was dissected into cerebellum, cerebrum, and brain stem. The cerebrum was subdivided into the right and left hemisphere according to the primary tumor (right) and contralateral side. For the Gd quantification, the tissue samples were manually homogenized. From each tissue, about 20 mg was weighed, mixed with 50 μL of 100 nM Tb(NO_3_)_3_ as an internal standard, and dried for 2 h at 95 °C. Subsequently, 50 μL of concentrated nitric acid (65% HNO_3_, Suprapur; Merck KgaA, Darmstadt, Germany) and 20 μL of hydrogen peroxide (H_2_O_2_, Emsure; Merck KgaA) were added, and samples were heated to dissolve the tissue for 2 h at 95 °C in a microwave oven (MDS 2000; CEM, Kamp-Lintfort, Germany). All samples were prepared in triplicate and were measured using ICP-MS (Agilent 7900; Waldbronn, Germany).

### Statistical evaluation

Statistical comparison between the three groups was done with the Kruskal-Wallis test. The calculations were performed with GraphPad Prism (GraphPad Software, La Jolla, CA, USA) using a significance level of 5%.

## Results

### Blood-brain barrier disruption in the tumor model

The GS9L tumor model was successfully established except for one animal, which died in anesthesia shortly after tumor cell inoculation. For the other 5 rats, the presence of the tumor and the consequent local disruption of the BBB was confirmed by contrast-enhanced MRI 1 week after inoculation (day 8). A clear tumor enhancement restricted to the right hemisphere of the cerebrum was visible in 2–5 slices 1 h after gadopentetate dimeglumine administration (Fig. 2). Radiotherapy had limited efficacy on GS9L tumor growth. At the end of the study, the tumor was still present in the 5 animals. The tumor was larger than on day 8 and all animals showed a midline shift and an infiltration of the left hemisphere could not be excluded.

### MRI signal enhancement of the DCN

**Fig. 3:**
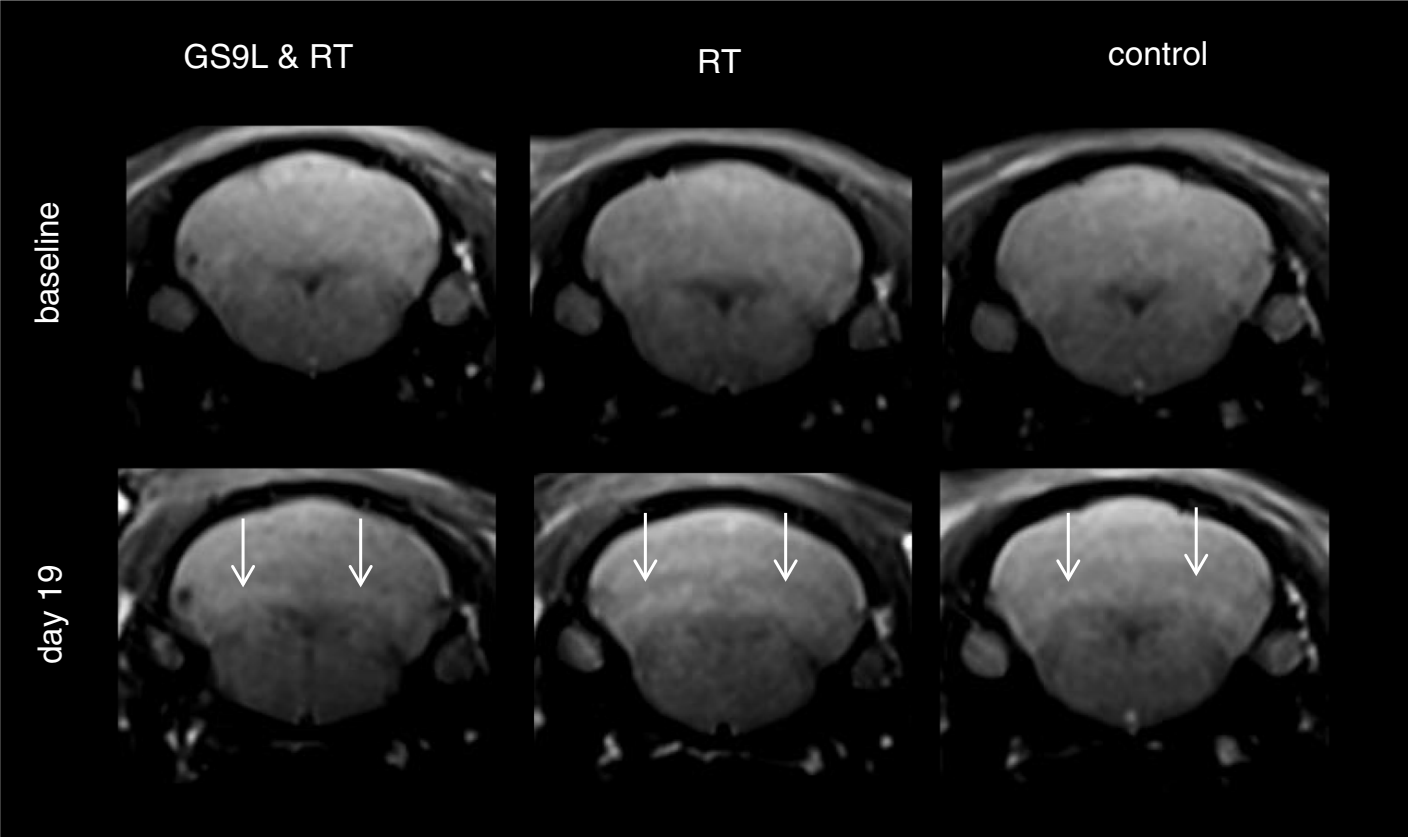
MR images of cerebellum including deep cerebellar nuclei before (baseline) and 1 week after administration of gadopentetate dimeglumine (day 19) for tumor-bearing animals (GS9L and RT), healthy animals that received radiotherapy (RT), and healthy animals without treatment (control). Enhanced cerebellar nuclei are indicated by arrows

**Fig. 4:**
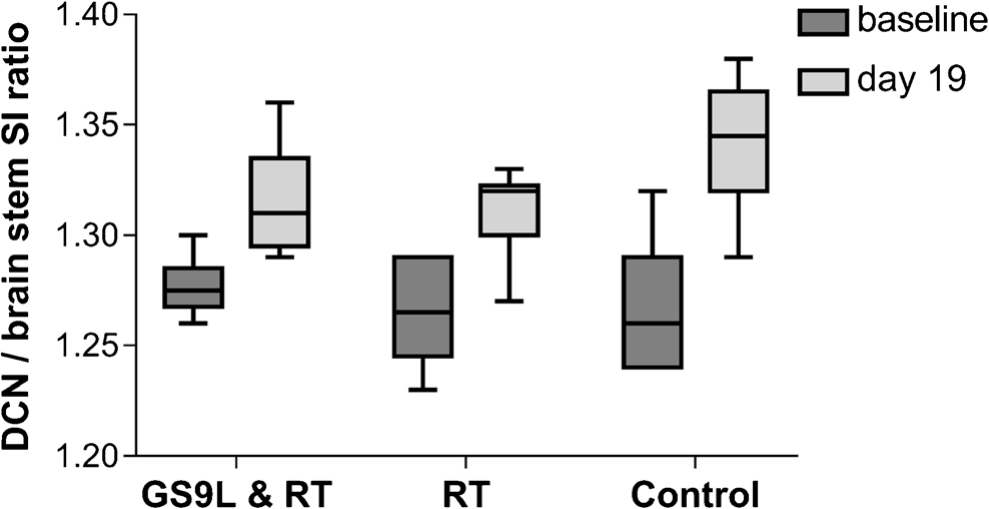
Quantitative MRI signal intensity (SI) evaluation. The SI ratio of deep cerebellar nuclei (DCN) to brain stem before (baseline) and 1 week after repeated administration of gadopentetate dimeglumine (day 19) for tumor-bearing animals (GS9L+RT), healthy animals that received radiotherapy (RT), and healthy animals without treatment (control)

Representative images of the cerebellum containing the DCN for each experimental group before and after repeated GBCA administration are shown in Fig. 3. Visually evident enhancements of the DCN were observed for all groups 1 week after the last injection. The quantitative analysis of the DCN/brain stem SI ratio is summarized in Fig. 4. One week after the last GBCA injection, higher SI ratios were found in the tumor group, the radiotherapy group, and the control group compared with the respective values of the baseline MRI. Neither radiotherapy alone nor the combination of the GS9L tumor and radiotherapy had an impact on SI ratios. Between the three groups, no significant differences in the SI ratios were found at baseline (*p* = 0.59) and 1 week after the last administration (*p* = 0.14).

### Brain Gd concentration

**Fig. 5:**
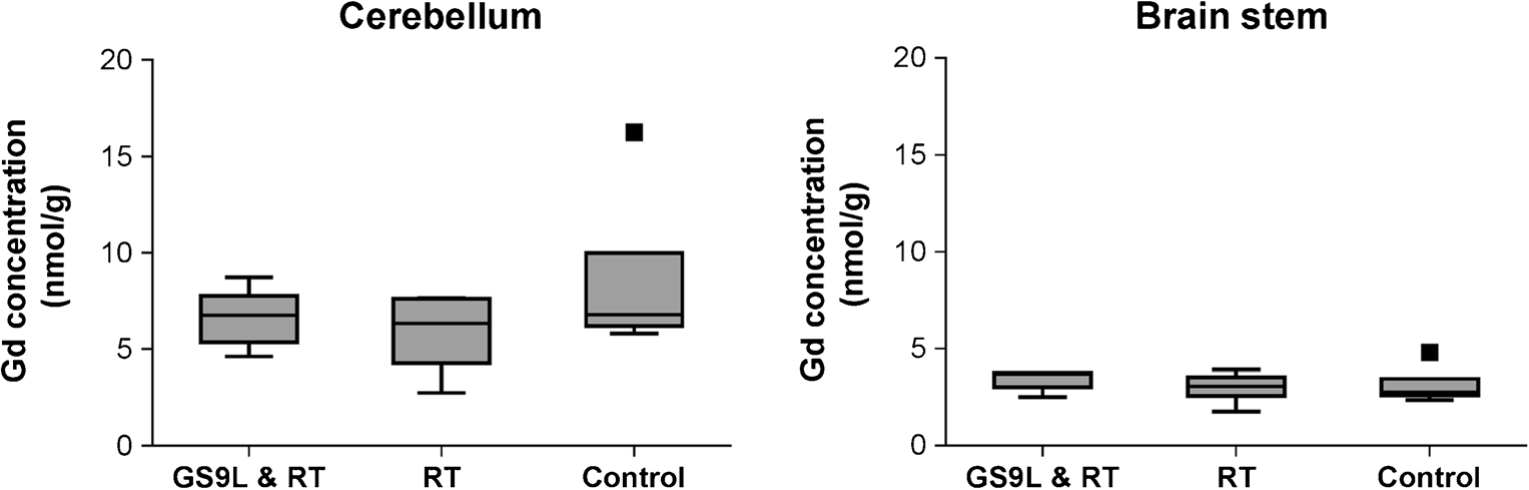
Gadolinium (Gd) concentrations in the cerebellum (left) and brain stem (right) 1 week after repeated administration of gadopentetate dimeglumine for tumor-bearing animals (GS9L+RT), healthy animals that received radiotherapy (RT), and healthy animals without treatment (control)

The Gd concentrations found in brain regions unaffected by the tumor model (cerebellum and brain stem) were not increased by radiotherapy or the combination of tumor and radiotherapy. The median Gd concentrations in the cerebellum 1 week after the last injection were 6.7 nmol/g (GS9L and radiotherapy group), 6.3 nmol/g (radiotherapy group), and 6.8 nmol/g (control group). In the brain stem, 1.8- to 2.4-fold lower concentrations with 3.7 nmol/g (GS9L and RT), 3.1 nmol/g (RT), and 2.8 nmol/g (control) were detected (Fig. 5). No significant differences were found between the three groups (*p* = 0.64 for cerebellum, *p* = 0.4 for brain stem).

**Fig. 6:**
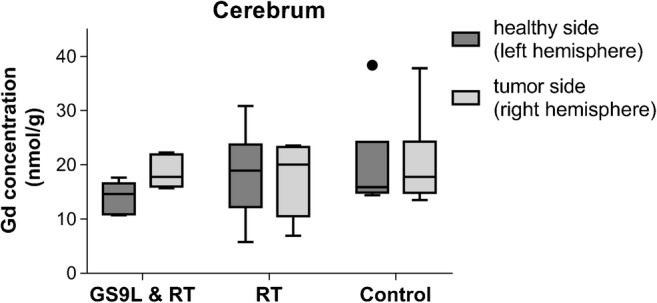
Gadolinium (Gd) concentrations in the cerebrum for the left and right hemisphere 1 week after repeated administration of gadopentetate dimeglumine for tumor-bearing animals (GS9L and RT), healthy animals that received radiotherapy (RT), and healthy animals without treatment (control). The tumor was inoculated in the right hemisphere

For the cerebrum, the Gd concentration was quantified separately for the tumor-bearing right and the left hemisphere. The medians of the respective concentrations were 17.8/14.6 nmol/g (GS9L and radiotherapy), 20.0/18.9 nmol/g (radiotherapy), and 17.8 / 15.9 nmol/g (control) for the right and left hemisphere respectively (Fig. 6). In the GS9L and radiotherapy group, the median Gd content in the primary tumor side was 22% higher than in the contralateral hemisphere. The differences between hemispheres in the radiotherapy (6%) and control group (12%) were less pronounced. No significant different Gd concentrations between the three groups were observed for the right (*p* = 0.98) and for the left (*p* = 0.41) hemisphere.

## Discussion

In this study, the impact of a local BBB disruption on MRI signal changes in the cerebellar nuclei and the presence of gadolinium in the brain after repeated GBCA injection were investigated in an experimental setting. Therefore, a group of tumor-bearing rats treated with radiotherapy was compared with healthy rats that underwent radiotherapy and a group of untreated control rats. All animals received 5 injections of gadopentetate dimeglumine, a linear GBCA that had induced MRI signal hyperintensities in the cerebellar nuclei in previous clinical and preclinical studies [1, 3, 4, 13]. In our study, the disruption of the BBB had no impact on DCN hyperintensity. No significant differences of the DCN/brain stem SI ratio were found among the three groups. Furthermore, the similar concentrations of Gd detected in the brain for all groups confirmed the MRI analysis and demonstrated that irradiation damage alone or a tumor-induced local BBB disruption in combination with irradiation does not significantly alter the amount of Gd present in the brain 1 week after repeated GBCA administration. That indicates that GBCA penetrating the tumor environment is rapidly eliminated and does not contribute to the observed Gd brain retention, which seems to be independent of the BBB integrity.

Our results are partly in contrast to other preclinical data. In a chronic hypoperfusion brain model in rats that is associated with BBB alterations, repeated administration of linear gadodiamide (cumulative dose 13.2 mmol/kg) led to higher T1-weighted SIs in the DCN and higher Gd concentrations in the cerebellum and subcortical region [28]. Although the Gd concentrations in the hypoperfused animals were significantly higher compared with the control animals, they differ not substantially (on average 1.2-fold and 1.3-fold for cerebellum and subcortical region, respectively). In addition, no difference was found in the cortical brain. It needs to be pointed out that the hypoperfusion brain model does not only alter BBB integrity but also reduces cerebral blood flow. This can also impact the GBCA kinetics in the brain.

Another recently published animal study investigated the impact of brain inflammation on the Gd brain retention after repeated administration of gadopentetate dimeglumine (cumulative dose 20 mmol/kg) in an experimental autoimmune encephalomyelitis (EAE) mouse model [29]. Although the small sample size (*n* = 2/group and time-point) does not allow a quantitative analysis, clearly (about twofold) higher concentrations in the EAE brains compared with the controls were found. However, the influence of the BBB alteration on the brain Gd content is not clear as the authors mentioned that also the blood-CSF barrier is altered already during the early phases of EAE. They hypothesized that inflammation-mediated glycosaminoglycan alteration may promote the higher Gd retention in tissue [29].

There are only few clinical data available that addresses the impact of BBB alterations on brain Gd amount. McDonald et al. evaluated Gd concentrations in postmortem brains of two patient groups. One group underwent contrast-enhanced MRI for CNS pathologies, mostly metastases and glioblastoma [30], and the other group had no intracranial abnormalities and had contrast-enhanced MRI for non-neuropathological indications [31]. They found a comparable fraction of the administered Gd dose present in most neuroanatomic regions between patients with and without intracranial abnormalities. Interestingly, they report a sixfold higher Gd retention in the globus pallidus of patients with normal brain morphology compared with patients with neuropathologies [31]. However, as the availability of postmortem brain tissue is scarce, the group size in these studies is very small and different. Furthermore, the patient population is heterogeneous with regard to GBCA dose and time interval between MRI and death which limits conclusions. Nonetheless, these data point towards Gd presence independent of BBB integrity in a clinical setting.

In our study, we used an orthotopic rat gliosarcoma tumor model [32]. The local disruption of the BBB was confirmed by the contrast enhancement of the tumor on the MRI after the first gadopentetate dimeglumine injection. The tumor-bearing animals underwent a fractionated radiotherapy treatment regimen [33] to reduce progressive tumor growth in order to prolong the time interval that is needed between verification of BBB disruption, repeated GBCA injection, acute phase GBCA elimination, and readout (MRI, ICP-MS). One week after the last GBCA injection, the tumor on the T1-weighted images showed clear signs of progressive grow (lesion size increase, midline shift, and potential contralateral infiltration). This indicates that the BBB disruption was still present or was at least persistent for a longer time after the control MRI on day 8. One week after the last GBCA injection, the local Gd concentration at the site of tumor inoculation (right cerebrum) was on average ~ 20% higher than on the contralateral site. However, the concentrations did not significantly differ compared with the radiotherapy and the control group. Interestingly, the Gd concentrations in the cerebrum of the GS9L and radiotherapy group showed a considerable lower variation compared with the other two groups. One might hypothesize that the tumor induced locally disrupted BBB allows both an infiltration of GBCA from blood into the tissue as well as an efficient elimination. However, that cannot be evaluated with the present data. The healthy animals that underwent only radiotherapy (radiotherapy group) and the animals from the control group showed very similar Gd concentrations and interindividual variations demonstrating that the used radiotherapy regimen alone did not affect the presence of Gd.

Our study supports the hypothesis that the initial pathway of GBCAs from the blood into the brain is the blood-CSF barrier followed by an intracerebral distribution and elimination by the glymphatic system [34–36]. This pathway seems to be independent of the BBB status as presence of the GS9L tumor did not alter the amount of Gd present in the brain 1 week after repeated GBCA administration. However, the tumor model is associated with a local BBB disruption which is different to inflammation, infection, or systemic treatments that may lead to a more general alteration of BBB permeability. In addition, the exact exchange mechanisms of fluids and substances among different barrier systems and brain compartments are complex [37]. The BBB, the blood-CSF barrier, and the glymphatic system are closely related and pathological processes affect their interaction [38]. Thus, the exact role of the BBB integrity on the distribution, elimination, and retention of GBCAs within the brain remains to be elucidated.

Our study has certain limitations. An experimental tumor model can approach but not replicate the conditions in brain tumor patients. Also, the used irradiation technique with X-ray energies in the kV-range, an untargeted radiation field, and a 6 × 5 Gy fractionation scheme differ from clinical radiation therapy. The GBCA dosing was adapted based on the recommended body surface normalization between rats and humans [39]. The used dose per injection of 1.8 mmol/kg corresponds to a human dose of 0.3 mmol/kg. The cumulative dose of 9 mmol/kg is considerably higher than in humans but lower than in the discussed animal studies using a total dose of 13.2 mmol/kg [28] and 20 mmol/kg [29]. In our study, MRI and dissection were done at a single time-point 1 week after the last GBCA administration. This does not allow conclusions about the impact of the brain tumor and radiotherapy on the long-term retention as previous studies demonstrate an ongoing elimination of gadopentetate dimeglumine from the healthy rat brain until 5 weeks after the last injection [13, 15].

In conclusion, the presence of a brain tumor treated by radiotherapy or radiotherapy alone did not alter the amount of Gd that was found in the rat brain and the MRI signal hyperintensity in the deep cerebellar nuclei 1 week after repeated administration of gadopentetate dimeglumine. These data suggests that a local blood-brain barrier disruption does not affect the amount of Gd present in the brain.
